# The effect of endurance and endurance-strength training on body composition and cardiometabolic markers in abdominally obese women: a randomised trial

**DOI:** 10.1038/s41598-021-90526-7

**Published:** 2021-06-11

**Authors:** Małgorzata Jamka, Edyta Mądry, Patrycja Krzyżanowska-Jankowska, Damian Skrypnik, Monika Szulińska, Radosław Mądry, Aleksandra Lisowska, Gulnara Batyrova, Monika Duś-Żuchowska, Anna Gotz-Więckowska, Paweł Bogdański, Jarosław Walkowiak

**Affiliations:** 1 Department of Pediatric Gastroenterology and Metabolic Diseases, Poznan University of Medical Sciences, Szpitalna Str. 27/33, 60-572 Poznań, Poland; 2 Department of Physiology, Poznan University of Medical Sciences, Święcickiego Str. 6, 60-781 Poznań, Poland; 3 Department of Treatment of Obesity, Metabolic Disorders and Clinical Dietetics, Poznan University of Medical Sciences, Szamarzewskiego Str. 82, 60-569 Poznań, Poland; 4 Department of Oncology, Poznan University of Medical Sciences, Szamarzewskiego Str. 84, 60-569 Poznań, Poland; 5 Department of Clinical Auxology and Pediatric Nursing, Poznan University of Medical Sciences, Szpitalna Str. 27/33, 60-572 Poznań, Poland; 6 Department of Laboratory and Visual Diagnostics, West Kazakhstan Marat Ospanov Medical University, Maresyev Str. 68, Aktobe, 030019 Kazakhstan; 7 Department of Ophthalmology, Poznan University of Medical Sciences, Szamarzewskiego Str. 84, 60-569 Poznań, Poland

**Keywords:** Obesity, Lifestyle modification, Weight management

## Abstract

Studies comparing the effect of endurance and endurance-strength training on cardiometabolic markers provided inconsistent results. Therefore, the study aimed to compare the effect of endurance and endurance-strength training on body composition and cardiometabolic parameters in abdominally obese women. In this randomised trial, 101 subjects were included and divided into endurance (n = 52) and endurance-strength (n = 49) training. During the 12-week intervention, participants performed supervised one-hour training three times a week. Body composition, blood pressure (BP), markers of glucose and lipid homeostasis, and myoglobin levels were measured before and after the intervention. In total, 85 subjects completed the trial. Both interventions decreased fat mass and visceral adipose tissue and increased free fat mass, appendicular lean mass index and lean mass index. Neither endurance training nor endurance-strength training affected glucose and lipid metabolism. However, only endurance training significantly decreased paraoxonase and myoglobin levels. Both training programmes significantly decreased BP, with a more reduction of diastolic BP noted in the endurance group. In conclusion, both training programmes had a favourable effect on body composition but did not improve glucose and lipid homeostasis. Besides, endurance training decreased paraoxonase activity and myoglobin levels and was more effective in reducing BP.

The study was registered with the German Clinical Trials Register (DRKS) within the number: DRKS00019832 (retrospective registration), date of registration: 26/02/2020.

## Introduction

According to the World Health Organization (WHO), abdominal obesity (also known as central obesity) is defined as a waist circumference of more than 80 cm in women and 94 cm in men or a waist-to-hip ratio (WHR) of more than 0.85 and 0.90 in women and men, respectively^1^. This type of obesity is an independent risk factor for cardiovascular diseases, dyslipidaemia, hypertension, type 2 diabetes mellitus and impaired glucose tolerance. It also predisposes to several types of cancers^1,2^. It should be noted that this risk increases with a higher amount of abdominal fat^3^ and obesity also results in a higher risk of general mortality^1,4^. Furthermore, the results of the Framingham Heart Study showed that excessive body weight at the age of 40 reduces life expectancy by around three years^5^.

Physical activity provides numerous benefits for obese subjects. Together with diet, exercises play an important role in the primary prevention and management of excessive body weight^6–9^ mostly due to favourable impact on body composition, prevention of obesity-related diseases and improve cardiometabolic parameters^10–12^. Therefore, the American College of Sports Medicine^13,14^, the European College of Sport Science^15^ and the American Heart Association^8^ recommend a minimum of 30 min of moderate-intensity endurance training five days per week or a minimum of 20 min of vigorous endurance activity three days per week. Besides, regular strength training with eight to twelve repetitions for at least two days per week is also recommended.

Several meta-analyses have shown a significant effect of both endurance and strength training on anthropometric and cardiometabolic parameters, providing evidence for reductions in body weight, body mass index (BMI), waist circumference, fat mass (FM), improved lipid profile, decreased glucose, insulin levels and blood pressure (BP)^16–20^. Although the benefits of endurance and strength training alone are well documented, studies comparing the effect of endurance and endurance-strength training on body composition and cardiometabolic markers have proved inconsistent. While some studies reported that combined training is more effective than endurance training alone^21–23^, other studies did not find differences between the effects of both types of training^24,25^. Moreover, a previous meta-analysis which compared the effect of endurance, strength and combined training (including studies with both similar or longer duration than endurance or strength training alone) in overweight and obese subjects showed that endurance-strength training significantly increased lean body mass compared to endurance training. However, no other differences were observed between endurance and endurance-strength training^26^. As was reported previously, the effect of the exercise intervention on body composition and cardiometabolic markers may significantly differ between men and women^22,27–29^. Moreover, the effect of training may differ between pre- and postmenopausal women^30^. It is well known that menopause is linked to an increased risk number of health conditions, including cardiovascular diseases^31^. Besides, it has been shown that men of 70 years of age have lower cardiovascular risk as compared with women at age 50 (the median age of menopause^32^)^33,34^. Taking into account the negative effect of estrogen decline on the risk of cardiovascular diseases, we assume that women of perimenopausal age merit special attention. Therefore, this study aimed to assess the effect of endurance and endurance-strength training on body composition and cardiometabolic parameters in women aged 50–60 years with abdominal obesity. We hypothesised that there are no differences between the effect of endurance and endurance-strength training on body composition and cardiometabolic parameters in women with abdominal obesity. However, we believe that the training intervention in this age group may prevent further deterioration of health in women. We also hope that our findings help to improve women’s health through the promotion of endurance-strength training in this group.

## Results

### Participants flow

**Figure 1:**
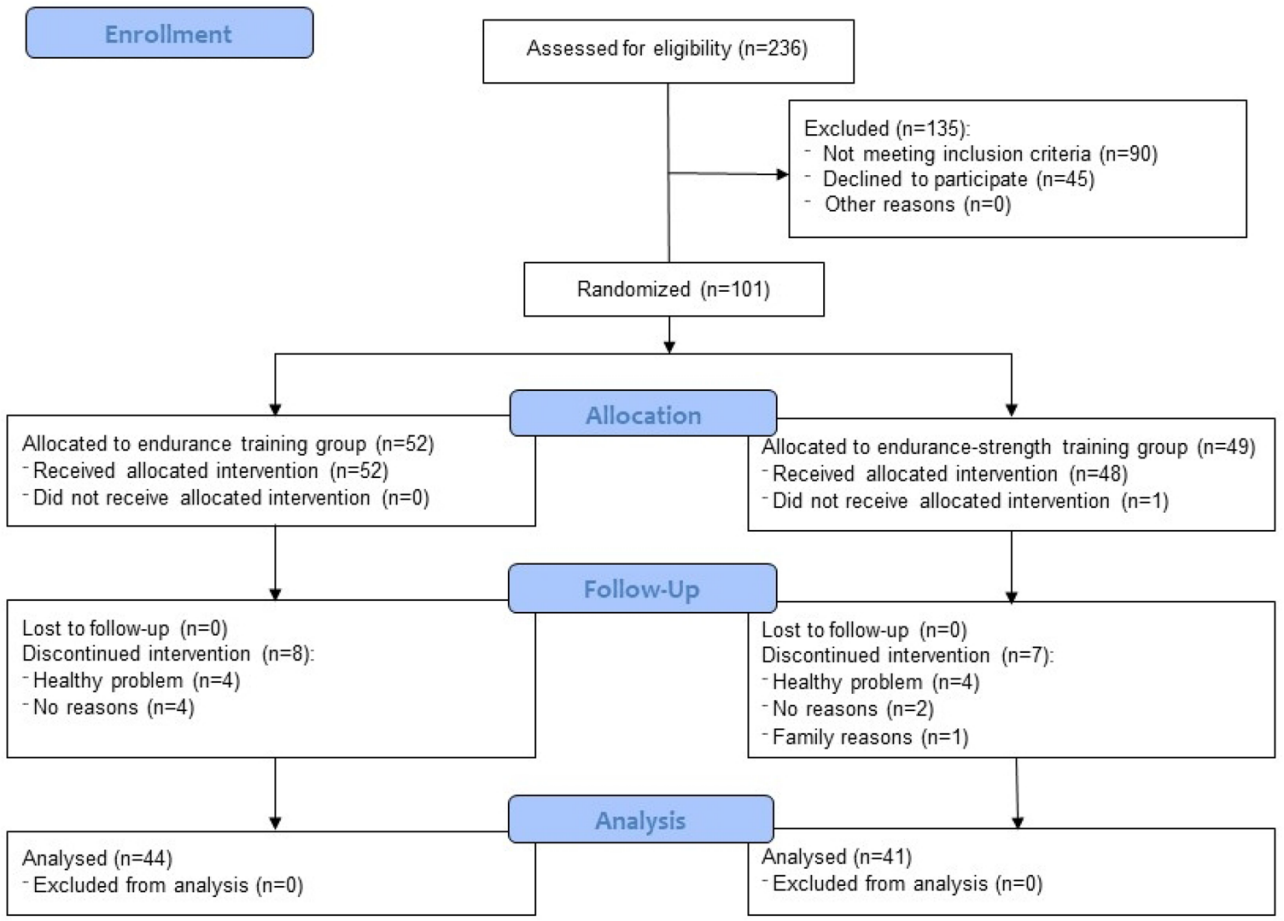
CONSORT 2010 flow diagram^83^. The Figure was previously published in the *Journal of Clinical Medicine* which publishes articles under an open access Creative Common CC BY license.

Volunteers were recruited to the study between January and August 2016, while the intervention was performed in two parts: the first started in April 2016 and finished in June 2016 (n = 48) and the second was performed between September and November 2016 (n = 53). Participant flow through the study is presented in Fig. 1. Out of 236 subjects assessed for eligibility, 90 were excluded because of not meeting the inclusion criteria and 45 subjects declined to participate. Out of the remaining subjects, 52 were randomised to the endurance training group and 49 were assigned to the endurance-strength training group. Only one subject from the endurance-strength training did not start allocated intervention. Eight subjects from endurance training and seven from endurance-strength training discontinued the intervention (eight due to health problems, six did not provide reasons but had low adherence to the intervention and one due to family reasons). A total of 85 postmenopausal women (44 for the endurance group and 41 for the endurance-strength training group) were included in the final analysis. The mean adherence was 91% and no differences between groups were observed. Besides, no significant side effects occurred. Six subjects reported a problem with joints or muscles, two subjects observed high BP and in one subject swelling was noted. Tables 1 and 2 summarise the baseline demographic and clinical characteristics of the study population. There were no statistically significant differences between groups at baseline.

**Table 1:** Anthropometric characteristics of the study population (n = 101).

		Endurance (n = 52)		Endurance-strength (n = 49)		*p*
		Median (Q1–Q3)	Mean ± SD (95% CI)	Median (Q1–Q3)	Mean ± SD (95% CI)	
**Anthropometric parameter**						
Age [years]		55 (50– 60)	55 ± 7 (53 to 57)	54 (50– 60)	55 ± 7 (53 to 58)	0.8358
Weight [kg]		93.4 (84.9– 104.9)	96.0 ± 15.1 (91.7 to 100.2)	91.0 (82.4– 101.8)	93.2 ± 13.9 (89.2 to 97.2)	0.4129
BMI [kg/m^2^]		35.64 (32.07– 38.00)	35.87 ± 4.43 (34.63 to 37.10)	35.42 (31.79– 39.10)	35.98 ± 5.10 (34.52 to 37.45)	0.8556
Waist circumference [cm]		109.0 (103.5– 114.0)	110.0 ± 10.1 (107.2 to 112.8)	108.0 (103.0–117.0)	109.9 ± 10.2 (106.9 to 112.8)	0.9973
Hip circumference [cm]		120.0 (116.0– 126.5)	121.6 ± 9.6 (118.9 to 124.2)	120.0 (113.0–127.0)	121.0 ± 11.3 (117.7 to 124.2)	0.5999
**Body composition by region**						
*Arms*	FM [g]	5447 (4538– 6400)	5680 ± 1557 (5247 to 6114)	5396 (4489– 6531)	5558 ± 1336 (5174 to 5942)	0.8207
	FFM [g]	4581 (4107–5379)	4801 ± 1097 (4495 to 5106)	4854 (4313–5387)	4963 ± 1116 (4642 to 5283)	0.3033
*Trunk*	FM [g]	20,810 (18,633– 23,883)	21,466 ± 4595 (20,187 to 22,746)	21,079 (17,308– 24,488)	21,350 ± 4722 (19,994 to 22,706)	0.9487
	FFM [g]	24,267 (22,302– 26,818)	25,257 ± 4244 (24,075 to 26,438)	25,165 (22,834–27,011)	25,385 ± 3403 (24,408 to 26,363)	0.5252
*Legs*	FM [g]	13,760 (122,260–15,555)	13,926 ± 2632 (13,194 to 14,659)	13,090 (10,793– 17,578)	14,226 ± 4148 (13,035 to 15,418)	0.7788
	FFM [g]	16,419 (14,699– 19,032)	18,320 ± 9553 (15,660 to 20,979)	16,668 (15,023–18,548)	16,984 ± 2842 (16,168 to 17,801)	0.9080
*Head*	FM [g]	1022 (946– 1063)	1016 ± 114 (984 to 1048)	1007 (926– 1109)	1023 ± 144 (981 to 1064)	0.8683
	FFM [g]	3437 (3264– 3627)	3472 ± 290 (3391 to 3553)	3525 (3286– 3771)	3540 ± 373 (3433 to 3647)	0.5386
*Total*	FM [g]	40,725 (37,046– 46,779)	41,988 ± 7767 (39,826 to 44,150)	41,134 (34,199–48,285)	42,222 ± 9446 (39,509 to 44,936)	0.9379
	FFM [g]	48,736 (44,280– 53,978)	50,645 ± 8130 (48,381 to 52,908)	50,572 (45,649– 54,524)	50,915 ± 6450 (49,063 to 52,768)	0.4861
*Male (android)*	FM [g]	3784 (3388– 4231)	3870 ± 889 (3622 to 4117)	3974 (2995– 4460)	3900 ± 1034 (3603 to 4197)	0.9272
	FFM [g]	3965 (3616– 4384)	4118 ± 789 (3899 to 4338)	3936 (3565–4443)	4133 ± 797 (3905 to 4362)	0.8491
*Female (gynoidal)*	FM [g]	6583 (5974– 7787)	6718 ± 1309 (6354 to 7082)	6702 (5710– 7732)	6726 ± 1600 (6266 to 7186)	0.7945
	FFM [g]	7901 (7239– 8715)	8116 ± 1340 (7743 to 8489)	7963 (7299–8690)	8136 ± 1104 (7819 to 8453)	0.7111
**Other**						
VAT [g]		1029 (889– 1236)	1062 ± 240 (995 to 1129)	1035 (826–1309)	1078 ± 320 (986 to 1170)	0.8207
LMI [kg/m^2^]		17.6 (116.6–19.3)	18.2 ± 2.1 (17.6 to 18.7)	17.9 (17.4– 19.3)	18.5 ± 1.9 (18.0 to 19.1)	0.2889
ALMI [kg/m^2^]		7.62 (7.00– 8.29)	7.76 ± 1.05 (7.46 to 8.05)	7.62 (7.30–8.40)	7.95 ± 0.98 (7.67 to 8.23)	0.3731

*ALMI* appendicular lean mass index, *BMI* body mass index, *FFM* free fat mass, *FM* fat mass, *LMI* lean mass index, *VAT* visceral adipose tissue.

**Table 2:** Metabolic characteristics of the study population (n = 101).

	Endurance (n = 52)		Endurance-strength (n = 49)		*p*
	Median (Q1–Q3)	Mean ± SD (95% CI)	Median (Q1–Q3)	Mean ± SD (95% CI)	
**Glucose homeostasis**					
Glucose [mg/dl]	95 (90–103)	98 ± 12 (95 to 101)	97 (90–103)	99 ± 15 (95 to 103)	0.6086
Insulin [µU/ml]	13.3 (8.9–17.2)	14.4 ± 6.8 (12.5 to 16.3)	13.6 (8.3–18.4)	15.2 ± 8.8 (12.7 to 17.8)	0.5678
HbA1c [%]	5.5 (5.2–5.7)	5.5 ± 0.4 (5.4 to 5.6)	5.5 (5.3–5.7)	5.6 ± 0.4 (5.4 to 5.7)	0.9158
IGF-1 [ng/ml]	119.61 (97.74–138.61)	124.35 ± 36.18 (114.28 to 134.42)	113.59 (92.96–129.53)	113.56 ± 29.10 (105.20 to 121.92)	0.2172
HOMA-IR	3.01 (2.20–4.18)	3.53 ± 1.90 (3.00 to 4.06)	3.17 (1.92–4.92)	3.81 ± 2.40 (3.13 to 4.50)	0.7962
QUICKI	0.55 (0.51–0.59)	0.55 ± 0.06 (0.53 to 0.57)	0.54 (0.49–0.61)	0.55 ± 0.09 (0.53 to 0.58)	0.7962
**Lipid homeostasis**					
TC [mg/dl]	197 (170–236)	207 ± 47 (194 to 220)	218 (190–235)	214 ± 34 (204 to 224)	0.4485
LDL-C [mg/dl]	114 (96–146)	120 ± 40 (109 to 131)	126 (105–144)	125 ± 29 (117 to 133)	0.6325
HDL-C [mg/dl]	54 (45–67)	57 ± 16 (53 to 62)	60 (51–70)	60 ± 14 (56 to 64)	0.1111
TG [mg/dl]	125 (84–168)	143 ± 89 (118 to 168)	125 (95–163)	144 ± 71 (123 to 164)	0.8823
ox-LDL [ng/ml]	389 (223–1411)	793 ± 776 (577 to 1009)	277 (189–912)	655 ± 697 (455 to 856)	0.3277
ApoA1 [g/l]	1.65 (1.46–1.90)	1.66 ± 0.27 (1.58 to 1.73)	1.65 (1.53–1.95)	1.71 ± 0.26 (1.63 to 1.78)	0.4751
ApoB [g/l]	0.89 (0.77–1.16)	0.98 ± 0.28 (0.90 to 1.05)	0.98 (0.83–1.14)	1.00 ± 0.22 (0.94 to 1.07)	0.2856
ApoB/ApoA1	0.59 (0.49–0.76)	0.63 ± 0.19 (0.57 to 0.68)	0.61 (0.50–0.73)	0.62 ± 0.16 (0.57 to 0.66)	0.9703
PON [U/l]	446.73 (353.13–508.50)	504.47 ± 260.56 (431.93 to 577.01)	392.00 (273.08–532.41)	465.74 ± 498.76 (322.48 to 609.00)	0.1430
Myoglobin [ng/ml]	31.6 (24.3–37.8)	35.3 ± 16.9 (30.6 to 40.0)	32.1 (23.0–43.6)	34.7 ± 15.2 (30.3 to 39.1)	1.0000
**Blood pressure**					
SBP [mmHg]	146 (133–158)	147 ± 17 (142 to 151)	143 (145–158)	146 ± 18 (141 to 151)	0.8757
DBP [mmHg]	86 (80–92)	86 ± 11 (83 to 89)	82 (77–89)	83 ± 14 (79 to 87)	0.0693

*ApoA1* apolipoprotein A1, *ApoB*, apolipoprotein B, *DBP* diastolic blood pressure, *HbA1c* glycated haemoglobin, *HDL-C* high-density lipoprotein cholesterol, *HOMA* homeostatic model assessment for insulin resistance, *IGF-1* insulin-like growth factor, *LDL-C* low-density lipoprotein cholesterol, *ox-LDL* oxidized low-density lipoprotein, *PON* paraoxonases, *SBP* systolic blood pressure, *TC* total cholesterol, *TG* triglycerides, *QUICKI* quantitative insulin sensitivity check index.

### The effect of endurance and endurance-strength training on body composition and cardiometabolic parameters

The effect of endurance and endurance-strength training on body composition is presented in Table 3. After the intervention period, we observed a decrease of visceral adipose tissue (VAT) and FM for total and individual parts of the body (except the head) and an increase of free fat mass (FFM), lean mass index (LMI) and appendicular lean mass index (ALMI) in both groups.

**Table 3:** Effects of endurance and endurance-strength training on body composition.

Body region		Endurance (n = 44)				*p*	Endurance-strength (n = 41)				*p*
		Pre-intervention		Post-intervention			Pre-intervention		Post-intervention		
		Median (Q1–Q3)	Mean ± SD (95% CI)	Median (Q1–Q3)	Mean ± SD (95% CI)		Median (Q1–Q3)	Mean ± SD (95% CI)	Median (Q1–Q3)	Mean ± SD (95% CI)	
**Arms**	FM [g]	5447 (4478–6529)	5703 ± 1636 (5206 to 6201)	5416 (4345–5980)	5381 ± 1316 (4981 to 5781)	0.0011	5396 (4484–6546)	5535 ± 1365 (5104 to 5966)	4824 (4255–5876)	5120 ± 1256 (4724 to 5517)	< 0.0001
	FFM [g]	4619 (4153–5324)	4864 ± 1008 (4557 to 5170)	4896 (4366–5366)	5066 ± 1103 (4731 to 5401)	0.0009	4661 (4283–5383)	4829 ± 933 (4535 to 5124)	4945 (4454–5476)	5022 ± 776 (4777 to 5267)	0.0281
**Trunk**	FM [g]	20,256 (18,633–23,780)	21,459 ± 4754 (20,014 to 22,904)	18,706 (17,018–22,734)	20,172 ± 4724 (18,736 to 21,608)	< 0.0001	20,773 (17,194–23,660)	20,663 ± 4151 (19,352 to 21,973)	18,598 (16,549–22,271)	19,437 ± 3982 (18,181 to 20,694)	< 0.0001
	FFM [g]	24,516 (22,073–26,818)	25,284 ± 4434 (23,936 to 26,632)	25,523 (23,395–28,160)	26,349 ± 4445 (24,998 to 27,701)	< 0.0001	24,965 (22,721–26,924)	24,984 ± 3000 (24,037 to 25,931)	25,357 (23,502–27,950)	25,864 ± 3531 (24,749 to 26,978)	0.0004
**Legs**	FM [g]	13,760 (12,455–15,555)	13,968 ± 2577 (13,185 to 14,752)	12,702 (11,418–14,518)	12,966 ± 2673 (12,154 to 13,778)	< 0.0001	13,045 (10,793–17,644)	14,167 ± 4156 (12,856 to 15,479)	12,076 (10,000–14,830)	12,909 ± 3891 (11,680 to 14,137)	< 0.0001
	FFM [g]	16,317 (14,742–18,721)	17,168 ± 3314 (16,160 to 18,175)	16,978 (15,123–18,750)	17,500 ± 3071 (16,566 to 18,434)	0.0157	16,668 (15,023–18,481)	16,776 ± 2601 (15,955 to 17,597)	16,708 (15,537–19,268)	17,435 ± 2655 (16,597 to 18,273)	0.0011
**Head**	FM [g]	1005 (921–1053)	1007 ± 119 (970 to 1043)	1005 (930–1055)	988 ± 91 (960 to 10,160	0.4274	1007 (920–1110)	1023 ± 152 (975 to 1071)	1005 (934–1086)	1024 ± 122 (985 to 1062)	0.3888
	FFM [g]	3401 (3213–3597)	3430 ± 280 (3344 to 3515)	3455 (3232–3588)	3432 ± 269 (3351 to 3514)	0.9535	3525 (3286–3830)	3567 ± 394 (3443 to 3692)	3568 (3299–3834)	3602 ± 425 (3467 to 3736)	0.1504
**Total**	FM [g]	40,629 (37,046–45,894)	42,069 ± 7913 (39,664 to 44,475)	38,324 (35,215–43,207)	39,507 ± 7513 (37,223 to 41,791)	< 0.0001	41,134 (33,653–47,563)	41,410 ± 885 (38,614 to 44,206)9	35,711 (31,535–45,235)	38,563 ± 8185 (23,980 to 41,147)	< 0.0001
	FFM [g]	48,901 (44,280–53,978)	50,788 ± 8382 (48,240 to 53,337)	50,409 (46,445–56,384)	52,330 ± 8167 (49,847 to 54,813)	< 0.0001	50,428 (45,649–52,690)	50,234 ± 5666 (48,445 to 52,022)	51,075 (47,774–55,820)	51,825 ± 6735 (49,699 to 53,950)	< 0.0001
**Male (android)**	FM [g]	3718 (3418–4231)	3866 ± 932 (3583 to 4150)	3386 (2964–3992)	3550 ± 911 (3273 to 3827)	< 0.0001	3664 (2982–4451)	3767 ± 917 (3478 to 4057)	3295 (2750–3993)	3444 ± 852 (3175 to 3713)	< 0.0001
	FFM [g]	3997 (3530–4384)	4119 ± 811 (3872 to 4365)	4114 (3592–4727)	4271 ± 827 (4019 to 4522)	0.0013	3865 (3541–4322)	4010 ± 699 (3789 to 4230)	4120 (3847–4609)	4214 ± 691 (3996 to 4432)	0.0003
**Female (gynoidal)**	FM [g]	6450 (5974–7632)	6693 ± 1309 (6295 to 7092)	6174 (5436–7194)	6234 ± 1262 (5851 to 6618)	< 0.0001	6661 (5710–7732)	6646 ± 1600 (6141 to 7151)	6067 (4857–7089)	6142 ± 1500 (5668 to 6615)	< 0.0001
	FFM [g]	7901 (7239–8715)	8118 ± 1372 (7701 to 8535)	8291 (7578–9420)	8511 ± 1334 (8106 to 8917)	< 0.0001	7854 (7299–8582)	8034 ± 982 (7724 to 8344)	8191 (7511–9187)	8407 ± 1123 (8052 to 8761)	0.0001
**Other**											
VAT [g]		1026 (887–1236)	1063 ± 248 (988 to 1139)	958 (807–1088)	978 ± 254 (901 to 1055)	< 0.0001	1020 (822–1242)	1053 ± 290 (961 to 1144)	899 (732–1058)	927 ± 269 (842 to 1012)	< 0.0001
LMI [kg/m^2^]		17.6 (16.7–19.3)	18.1 ± 2.1 (17.5 to 18.8)	18.3 (17.2–20.3)	18.7 ± 2.2 (18.0 to 19.3)	< 0.0001	17.8 (16.9–19.2)	18.3 ± 1.8 (17.7 to 18.8)	18.6 (17.3–19.7)	18.9 ± 2.1 (18.2 to 19.6)	0.0001
ALMI [kg/m^2^]		7.62 (7.09–8.16)	7.77 ± 1.02 (7.46 to 8.08)	7.81 (7.42–8.29)	7.95 ± 1.02 (7.64 to 8.26)	0.0009	7.62 (7.26–8.32)	7.85 ± 0.89 (7.57 to 8.13)	7.83 (7.54–8.53)	8.07 ± 1.00 (7.75 to 8.38)	0.0065

*ALMI* appendicular lean mass index, *FFM* free fat mass, *FM* fat mass, *LMI* lean mass index, *VAT* visceral adipose tissue.

The effect of endurance and endurance-strength training on glucose and insulin homeostasis, lipid metabolism and BP is shown in Table 4. None of the biochemical parameters analysed were affected by any of the training programmes except for paraoxonases (PON) activity and myoglobin levels which decreased in the endurance group. Fasting glucose and insulin levels, as well as glycated haemoglobin (HbA1c), insulin-like growth factor (IGF-1), the homeostatic model assessment for insulin resistance (HOMA) and the quantitative insulin sensitivity check index (QUICKI) did not change significantly. The lipid profiles (total cholesterol (TC), low-density lipoprotein cholesterol (LDL-C), high-density lipoprotein cholesterol (HDL-C), triglycerides (TG)) showed no significant changes after three months of intervention regardless of the training conditions. Besides, no significant changes were reported in oxidized low-density lipoprotein (ox-LDL), apolipoprotein A1 (ApoA1), apolipoprotein B (ApoB) levels, as well as ApoB/ApoA1 ratio. However, a significant decrease in systolic (SBP) and diastolic blood pressure (DBP) was found in both groups.

**Table 4:** Effects of endurance and endurance-strength training on metabolic parameters and blood pressure.

	Endurance (n = 44)				*p*	Endurance-strength (n = 41)				*p*
	Pre-intervention		Post-intervention			Pre-intervention		Post-intervention		
	Median (Q1–Q3)	Mean ± SD (95% CI)	Median (Q1–Q3)	Mean ± SD (95% CI)		Median (Q1–Q3)	Mean ± SD (95% CI)	Median (Q1–Q3)	Mean ± SD (95% CI)	
**Glucose homeostasis**										
Glucose [mg/dl]	96 (92–105)	99 ± 13 (95 to 103)	98 (94–108)	102 ± 16 (97 to 107)	0.1082	95 (89–103)	99 ± 15 (94 to 104)	98 (91–102)	99 ± 12 (95 to 103)	0.7332
Insulin [µU/ml]	13.3 (8.9–18.0)	14.7 ± 7.0 (12.6 to 16.9)	12.5 (9.6–18.6)	15.5 ± 10.4 (12.3 to 18.6)	0.5285	13.9 (9.8–19.0)	15.7 ± 8.4 (13.0 to 18.3)	12.1 (10.2–16.5)	15.3 ± 9.2 (12.4 to 18.2)	0.5678
HbA1c [%]	5.5 (5.3–5.8)	5.6 ± 0.4 (5.4 to 5.7)	5.5 (5.4–5.8)	5.6 ± 0.4 (5.5 to 5.7)	0.4043	5.6 (5.3–5.8)	5.6 ± 0.4 (5.5 to 5.7)	5.6 (5.5–5.8)	5.7 ± 0.3 (5.5 to 5.7)	0.2022
IGF-1 [ng/ml]	125.02 (102.89–142.46)	127.55 ± 35.78 (116.68 to 138.43)	129.78 (104.87–150.03)	129.30 ± 37.30 (117.96 to 140.65)	0.3688	118.10 (92.96–133.56)	115.80 ± 30.58 (106.15 to 125.46)	114.79 (97.28–135.41)	117.21 ± 27.65 (108.48 to 125.94)	0.6087
HOMA-IR	3.00 (2.25–4.23)	3.66 ± 1.99 (3.05 to 4.26)	3.05 (2.26–4.29)	4.04 ± 3.27 (3.05 to 5.03)	0.8519	3.23 (2.33–5.26)	3.88 ± 2.19 (3.19 to 4.57)	3.12 (2.38–3.97)	3.75 ± 2.35 (3.01 to 4.50)	0.5188
QUICKI	0.55 (0.51–0.59)	0.54 ± 0.06 (0.53 to 0.56)	0.55 (0.50–0.59)	0.55 ± 0.08 (0.53 to 0.58)	0.3269	0.54 (0.48–0.58)	0.54 ± 0.08 (0.52 to 0.57)	0.54 (0.51–0.58)	0.55 ± 0.07 (0.53 to 0.57)	0.6574
**Lipid homeostasis**										
TC [mg/dl]	200 (173– 241)	210 ± 48 (195 to 224)	202 (176–234)	209 ± 45 (196 to 223)	0.8473	213 (185–230)	210 ± 34 (199 to 221)	203 (186–223)	207 ± 34 (196 to 217)	0.0758
LDL-C [mg/dl]	115 (97–150)	124 ± 39 (112 to 136)	122 (103–147)	127 ± 37 (116 to 138)	0.6571	123 (102–142)	122 ± 30 (113 to 131)	119 (100–137)	121 ± 31 (111 to 131)	0.1992
HDL-C [mg/dl]	52 (44–65)	55 ± 14 (51 to 59)	53 (45–64)	55 ± 13 (51 to 59)	0.9004	60 (54–70)	61 ± 13 (57 to 65)	58 (51–67)	60 ± 12 (57 to 64)	0.4551
TG [mg/dl]	125 (87–168)	148 ± 93 (119 to 176)	118 (93–147)	134 ± 57 (117 to 151)	0.9750	116 (94–157)	134 ± 66 (113 to 155)	123 (94–149)	130 ± 50 (114 to 146)	0.8155
ox-LDL [ng/ml]	353 (204– 1368)	753 ± 758 (522 to 983)	292 (200–1287)	717 ± 737 (493 to 941)	0.0640	267 (174–1068)	697 ± 748 (461 to 933)	267 (160–1378)	730 ± 773 (486 to 974)	0.9090
ApoA1 [g/l]	1.64 (1.45–1.82)	1.64 ± 0.26 (1.56 to 1.72)	1.51 (1.40–1.87)	1.60 ± 0.27 (1.52 to 1.69)	0.1067	1.63 (1.53–1.92)	1.70 ± 0.25 (1.62 to 1.78)	1.69 (1.55–1.87)	1.70 ± 0.21 (1.64 to 1.77)	0.5188
ApoB [g/l]	0.89 (0.79–1.16)	0.99 ± 0.28 (0.91 to 1.07)	0.94 (0.77–1.07)	0.97 ± 0.27 (0.89 to 1.06)	0.1992	0.95 (0.82–1.14)	0.98 ± 0.22 (0.91 to 1.05)	0.96 (0.85–1.08)	0.95 ± 0.19 (0.90 to 1.02)	0.3747
ApoB/ApoA1	0.60 (0.51–0.76)	0.64 ± 0.19 (0.59 to 0.70)	0.58 (0.49–0.76)	0.64 ± 0.19 (0.58 to 0.70)	0.3231	0.59 (0.49–0.70)	0.60 ± 0.16 (0.56 to 0.65)	0.59 (0.52–0.65)	0.59 ± 0.14 (0.55 to 0.64)	0.3231
PON [U/l]	452.17 (374.00–523.00)	522.29 ± 275.60 (438.50 to 606.09)	363.82 (322.11–498.12)	469.67 ± 299.12 (378.73 to 560.61)	0.0196	392.00 (283.00–552.72)	490.76 ± 534.48 (322.05 to 659.46)	473.59 (327.00–593.08)	530.17 ± 545.17 (358.09 to 702.25)	0.1345
Myoglobin [ng/ml]	29.7 (24.1–35.6)	33.5 ± 16.8 (28.3 to 38.6)	27.2 (21.6–32.7)	29.0 ± 10.5 (25.7 to 32.1)	0.0065	30.8 (22.9–37.6)	33.2 ± 14.7 (28.5 to 37.8)	35.0 (23.6–44.0)	37.1 ± 18.2 (31.3 to 42.8)	0.1340
**Blood pressure**										
SBP [mmHg]	148 (132–159)	147 ± 17 (142 to 152)	137 (118–149)	135 ± 18 (130 to 141)	0.0010	143 (136–158)	147 ± 16 (142 to 152)	135 (128–143)	136 ± 14 (132 to 141)	0.0018
DBP [mmHg]	86 (80–93)	86 ± 11 (83 to 90)	77 (71–83)	77 ± 9 (73 to 79)	0.0001	83 (77–89)	84 ± 13 (80 to 88)	81 (75–85)	80 ± 8 (77 to 82)	0.0213

*ApoA1* apolipoprotein A1, *ApoB* apolipoprotein B, *DBP* diastolic blood pressure, *HbA1c* glycated haemoglobin, *HDL-C* high-density lipoprotein cholesterol, *HOMA* homeostatic model assessment for insulin resistance, *IGF-1* insulin-like growth factor, *LDL-C* low-density lipoprotein cholesterol, *ox-LDL* oxidized low-density lipoprotein, *PON* paraoxonases, *SBP* systolic blood pressure, *TC* total cholesterol, *TG* triglycerides, *QUICKI* quantitative insulin sensitivity check index.

### Comparison of the effect of endurance and endurance-strength training

Table 5 shows a comparison of the mean difference of changes in body composition and Table 6 presents the mean difference of changes in cardiometabolic parameters and BP between endurance and endurance-strength training using the ANCOVA test, adjusted for the baseline measures as a covariate. No differences between the effect of endurance and endurance-strength training on body composition were detected. Moreover, there were no differences between the effect of training programmes on fasting glucose and insulin levels, HbA1c, IGF-1, HOMA-IR and QUICKI as well as lipid profile and apolipoproteins levels. However, we showed significant differences in the effect of endurance and endurance-strength training on PON activity (mean (the 95% confidence interval of means (95% CI)):  − 52.63 (− 97.53 to  − 7.73) vs. 39.42 (− 15.53 to 94.36) U/l, *p* = 0.0287) and myoglobin levels (mean (95% CI):  − 4.3 (− 7.9 to  − 0.8) vs. 4.0 (− 0.5 to 8.5) ng/ml, *p* = 0.0028). Furthermore, no significant differences were found for SBP, whereas we observed a more significant reduction in DBP in the endurance group compared to the endurance-strength group (mean (95% CI):  − 9 (− 12 to  − 6) vs.  − 4 (− 7 to 0) mmHg, *p* = 0.0114).

**Table 5:** Comparison of the mean difference of changes in anthropometric parameters and body composition between endurance and endurance − strength training using the ANCOVA test, adjusted for the baseline measures as a covariate.

Body region		Endurance (n = 44)		Endurance-strength (n = 41)		*p*
		Median (Q1–Q3)	Mean ± SD (95% CI)	Median (Q1–Q3)	Mean ± SD (95% CI)	
**Arms**	Δ FM [g]	− 320 (− 564–4)	− 292 ± 1727 (− 466 to − 122)	− 415 (− 655 to − 201)	− 393 ± 1809 (− 556 to − 233)	0.2517
	Δ FFM [g]	147 (− 30–402)	170 ± 620 (68 to 278)	75 (− 70–375)	122 ± 566 (− 27 to 285)	0.5669
**Trunk**	Δ FM [g]	− 1441 (− 1939 to − 470)	− 1287 ± 1509 (− 1746 to − 828)	− 1250 (− 2186 to − 293)	− 1225 ± 1669 (− 1752 to − 698)	0.9976
	Δ FFM [g]	899 (143–1562)	1066 ± 1360 (652 to 1479)	868 (56–1873)	879 ± 1359 (450 to1308)	0.5293
**Legs**	Δ FM [g]	− 957 (− 1612 to − 371)	− 1002 ± 1026 (− 1314 to − 690)	− 1201 (− 2020 to − 471)	− 1259 ± 1211 (− 1641 to − 877)	0.0155
	Δ FFM [g]	439 (− 281–962)	334 ± 2612 (− 3 to 670)	578 (40–1369)	661 ± 2294 (212 to 1110)	0.2833
**Head**	Δ FM [g]	− 4 (− 47–31)	− 8 ± 175 (− 31 to 15)	5 (− 22–40)	7 ± 208 (− 11 to 24)	0.3000
	Δ FFM [g]	10 (− 83–77)	11 ± 151 (− 45 to 26)	10 (− 30–64)	7 ± 208 (− 11 to 24)	0.1371
**Total**	Δ FM [g]	− 2211 (− 3547 to − 1515)	− 2563 ± 2127 (− 3209 to − 1916)	− 2274 (− 4489 to − 1386)	− 2847 ± 2640 (− 3680 to − 2014)	0.4717
	Δ FFM [g]	1202 (683–2506)	1542 ± 1581 (1061 to 2022)	1488 (284– 3374)	1591 ± 2080 (934 to 2247)	0.9103
**Male (android)**	Δ FM [g]	− 269 (− 500 to − 80)	− 316 ± 317 (− 412 to − 220)	− 372 (− 487 to − 111)	− 323 ± 313 (− 422 to − 245)	0.7924
	Δ FFM [g]	180 (24–310)	150 ± 550 (52 to 248)	147 (64–419)	202 ± 517 (64 to 419)	0.5172
**Female (gynoidal)**	Δ FM [g]	− 446 (− 871 to − 169)	− 476 ± 1376 (− 618 to − 331)	− 469 (− 822 to − 158)	− 529 ± 1338 (− 690 to − 364)	0.5693
	Δ FFM [g]	504 (114–618)	394 ± 591 (278 to 510)	217 (88–717)	375 ± 418 (201 to 549)	0.8560
**Other**						
Δ VAT [g]		− 80 (− 149 to − 11)	− 85 ± 134 (− 126 to − 44)	− 139 (− 223 to − 39)	− 125 ± 134 (− 167 to − 83)	0.1357
Δ LMI [kg/m^2^]		0.4 (0.3–0.8)	0.5 ± 0.6 (0.4 to 0.7)	0.6 (0.1–1.3)	0.6 ± 0.8 (0.3 to 0.9)	0.7566
Δ ALMI [kg/m^2^]		0.20 (− 0.03–0.37)	0.18 ± 0.35 (0.08 to 0.29)	0.21 (− 0.08–0.60)	0.22 ± 0.49 (0.06 to 0.37)	0.6668

*ALMI* appendicular lean mass index, *FFM* free fat mass, *FM* fat mass, *LMI* lean mass index, *VAT* visceral adipose tissue.

**Table 6:** Comparison of the mean difference of changes in cardiometabolic parameters and blood pressure between endurance and endurance-strength training using the ANCOVA test, adjusted for the baseline measures as a covariate.

	Endurance (n = 44)		Endurance-strength (n = 41)		*p*
	Median (Q1–Q3)	Mean ± SD (95% CI)	Median (Q1–Q3)	Mean ± SD (95% CI)	
**Glucose homeostasis**					
Δ Glucose [mg/dl]	1 (− 3–11)	3 ± 10 (0 to 6)	0 (− 5–5)	0 ± 8 (− 2 to 2)	0.1036
Δ Insulin [µU/ml]	− 0.5 (− 3.9–2.3)	0.5 ± 10.6 (− 2.5 to 1.7)	− 0.3 (− 3.7–2.3)	− 1.4 ± 10.7 (− 3.3 to 0.7)	0.5752
Δ HbA1c [%]	0.0 (− 0.1–0.2)	0.0 ± 0.5 (− 0.1 to 0.1)	0.0 (− 0.1–0.1)	0.1 ± 0.5 (0.0 to 0.1)	0.3579
Δ IGF − 1 [ng/ml]	4.40 (− 14.05–18.18)	1.75 ± 26.69 (− 6.36 to 9.86)	− 3.33 (− 13.89–20.41)	1.41 ± 25.01 (− 6.48 to 9.30)	0.4775
Δ HOMA − IR	− 0.17 (− 0.96–0.68)	− 0.12 ± 2.36 (− 0.67 to 0.51)	− 0.07 (− 1.05–0.51)	− 0.44 ± 2.44 (− 0.92 to 0.11)	0.4292
Δ QUICKI	0.00 (− 0.03–0.05)	0.00 ± 0.06 (− 0.01 to 0.03)	0.00 (− 0.03–0.04)	0.01 ± 0.05 (− 0.01 to 0.02)	0.8927
**Lipid homeostasis**					
Δ TC [mg/dl]	− 3 (− 14–15)	− 2 ± 46 (− 11 to 7)	− 6 (− 14–3)	− 4 ± 17 (− 9 to 1)	0.8296
Δ LDL − C [mg/dl]	0 (− 12–17)	0 ± 41 (− 9 to 10)	− 2 (− 11–4)	− 2 ± 49 (− 7 to 3)	0.6411
Δ HDL − C [mg/dl]	0 (− 4–3)	0 ± 17 (− 2 to 2)	0 (− 6–3)	− 1 ± 15 (− 3 to 2)	0.8136
Δ TG [mg/dl]	− 3 (− 24–22)	− 5 ± 111 (− 20 to 9)	− 3 (− 18–22)	− 2 ± 118 (− 12 to 15)	0.5632
Δ ox − LDL [ng/ml]	− 13 (− 61–13)	− 49 ± 611 (− 113 to 18)	− 2 (− 56–45)	20 ± 590 (− 61 to 104)	0.2342
Δ ApoA1 [g/l]	− 0.02 (− 0.17–0.06)	− 0.03 ± 0.17 (− 0.08 to 0.02)	0.01 (− 0.05–0.08)	0.00 ± 0.15 (− 0.05 to 0.05)	0.1306
Δ ApoB [g/l]	− 0.04 (− 0.11–0.05)	− 0.03 ± 0.29 (− 0.08 to 0.02)	− 0.02 (− 0.13–0.07)	− 0.03 ± 0.30 (− 0.07 to 0.01)	0.9781
Δ ApoB/ApoA1	− 0.02 (− 0.07–0.03)	− 0.02 ± 0.21 (− 0.05 to 0.02)	− 0.02 (− 0.07–0.04)	− 0.01 ± 0.09 (− 0.04 to 0.01)	0.6172
Δ PON [U/l]	− 61.17 (− 145.64–55.53)	− 52.63 ± 147.69 (− 97.53 to − 7.73)	70.17 (− 34.85–151.80)	39.42 ± 174.08 (− 15.53 to 94.36)	0.0287
Δ Myoglobin [ng/ml]	− 3.8 (− 7.6–1.8)	− 4.3 ± 39.0 (− 7.9 to − 0.8)	3.8 (− 5.0–10.2)	4.0 ± 36.0 (− 0.5 to 8.5)	0.0028
**Blood pressure**					
Δ SBP [mmHg]	− 12 (− 21 to − 1)	− 11 ± 15 (− 16 to − 7)	− 12 (− 24 to − 1)	− 10 ± 18 (− 16 to − 5)	0.8084
Δ DBP [mmHg]	− 10 (− 16 to − 2)	− 9 ± 26 (− 12 to − 6)	− 4 (− 11–4)	− 4 ± 25 (− 7 to 0)	0.0114

*ApoA1* apolipoprotein A1, *ApoB* apolipoprotein B, *DBP* diastolic blood pressure, *HbA1c* glycated haemoglobin, *HDL-C* high-density lipoprotein cholesterol, *HOMA* homeostatic model assessment for insulin resistance, *IGF-1* insulin-like growth factor, *LDL-C* low-density lipoprotein cholesterol, *ox-LDL* oxidized low-density lipoprotein, *PON* paraoxonases, *SBP* systolic blood pressure, *TC* total cholesterol, *TG* triglycerides, *QUICKI* quantitative insulin sensitivity check index.

## Discussion

These results showed that endurance and endurance-strength training had no differential effect on body composition and did not affect glucose and lipid homeostasis. However, there were significant differences between the effect of endurance and endurance-strength training on PON activity, myoglobin levels and DBP. In contrast to endurance-strength training, endurance training significantly decreased PON activity, reduced myoglobin levels and was more effective in reducing DBP.

We showed that both training programmes had a favourable effect on body composition. Both endurance and endurance-strength training significantly decreased VAT and FM as well as increased FFM, LMI and ALMI. Similar results were obtained in our previous pilot study conducted on a small group of obese women^35^. After three months of the intervention, we reported a significant reduction in total body fat and total FM in both groups, while total body lean mass and total FFM decreased only in the endurance-strength training group. Nevertheless, no significant differences were observed between the groups for the investigated parameters. The favourable effect of training on body composition was also reported by Sillanpaa et al*.*^36^, who compared the effects of endurance and strength training, both alone and in combination, in women aged 39–64 years. During the 21-week training period, both strength and endurance groups trained two times a week and the combined group trained two times a week for strength and two times a week for endurance. After the intervention, the researchers observed significant reductions in total body fat and percentage of body fat in both groups, accompanied by an increase in FFM in the strength group and the combined group. However, no statistical differences between the groups were noted. In contrast, several studies reported significant differences between the effect of both types of training on body composition^22,25,27,37–39^. Church et al*.*^37^ compared the effect of resistance training, aerobic training and combined aerobic and resistance training (all interventions had approximately equal time requirements) and found that the combination of endurance and strength training improved FM significantly more than endurance training alone in type 2 diabetic subjects. In a recent meta-analysis, Marzolini et al*.*^38^ demonstrated that combined training was more effective than endurance training alone for improving total FFM, percent body fat, trunk fat, upper and lower limb strength in subjects with coronary artery disease. However, this meta-analysis included studies that compared the effect of endurance and combined training of similar duration and also studies in which combined training required significantly more time than endurance training alone. Moreover, Rossi et al*.*^25^ found that similar volume endurance and combined training decreased core fat and increased FFM, but only combined training potentiated a reduction in the percentage of body fat in obese postmenopausal women. Park et al*.*^39^ investigated the effect of similar duration and frequency endurance and combined training and observed that combination exercises were more effective in decreasing subcutaneous fat visceral fat than endurance exercise, with lean body mass significantly increased only in the combined training group. Interestingly, Sanal et al*.*^22^ reported gender differences in the effect of both types of training on body compositions, observing that in men, adding strength exercises to endurance training was more effective in increasing the FFM of arms, trunk and whole body, while in women combined training was more effective in reducing FM of legs. Another study also suggested that exercise-induced a more pronounced reduction in body weight and FM in men than women^27^. The difference in body composition between men and women could partly explain the differences between these results^40^. The demographic differences and the various methods adopted to assess body composition may explain the difference between the study results.

Physical activity may improve glucose and insulin homeostasis due to the transient increase in glucose uptake by the large exercised muscle mass^41^. The possible mechanism also includes positive adjustment of post insulin components such as the density of insulin protein receptors, protein kinase B and glycogen synthesis and glucose transferor protein^42^. Nevertheless, in our study, unlike previous studies, none of the training programmes affected glucose and insulin homeostasis. Recently, Azarbayjani et al*.*^43^ observed that 12 weeks of endurance, strength and concurrent training in a group of sedentary men significantly decreased insulin levels and insulin resistance. In the study, the endurance group worked for 30 min at an intensity of 60–70% reserve heart rate, whereas the strength group performed three sets of 10 repetitions at 70% of one-repetition maximum. The combined programme performed endurance exercises at 60–70% of the heart reserve rate for 20 min and two sets of ten repetitions at 70% one-repetition maximum. Besides, the previous meta-analysis compared the effects of endurance, strength and combined exercise training (with no restrictions on the exercise modality, intensity, volume, and frequency) on insulin resistance markers in overweight or obese children and adolescents, showing that endurance exercises were associated with declines in fasting insulin levels and HOMA^44^. However, AbouAssi et al*.*^23^ reported that combined training (full endurance training plus full resistance training) resulted in greater improvements in insulin sensitivity, β-cell function, and glucose effectiveness than either endurance or strength training alone. Importantly, approximately 52% of the improvement in insulin sensitivity by combined training was retained 14 days after the last exercise training bout. Importantly, it should be noted that the superior effect of combined training to endurance or strength training alone was mostly reported in studies where the endurance-strength training was the additive combination of endurance and strength exercises^23,45^. By comparison, when both groups had approximately equal training times, there were no large differences between groups^37,46^. The observed differences between studies may be also attributed to different subject characteristics, diet, primary glucose and insulin levels and time of drawing blood sample following the termination of the exercise protocol. Besides, normal insulin sensitivity in most of the study participants before the intervention may partly explain no effect of the intervention on this parameter.

The reduction in cholesterol levels is the gold standard in the prevention of cardiovascular diseases^47^. In a meta-analysis of 170,000 participants, it was reported that reductions in LDL-C levels decreased the incidence of heart attacks and ischaemic strokes^48^. It has also been reported that subjects with elevated TC levels have approximately twice the cardiovascular disease risk of those with optimal levels^49^. The prevalence of elevated TC levels is especially high in Europe, where 54% of adults aged 25 years and older have TC levels above the recommendation^50^. Besides, it has been shown that exercises have a positive impact on the improvement of the lipid profile, however, the optimal type, frequency, intensity and duration of training for improvement of cholesterol levels have not yet been identified^47^. Among adults, a recent meta-analysis in subjects with type 2 diabetes suggested that combined training is the most efficacious to improve lipid profile compared with endurance or strength training alone^51^. In contrast, a previous meta-analysis conducted in overweight and obese adult subjects observed no significant differences for TC, LDL-C, HDL-C and TG between training programmes^26^. However, it should be highlighted that the meta-analyses did not precise if both training programmes had a similar or different volume^26,51^. Here, we observed no effect of the intervention on the lipid profile and detected no differences between study groups, suggesting that exercise did not meet the intensity needed to improve lipid profiles. However, in our previous pilot study, we applied the same volume, duration and intensity of training and showed increase TC levels in both groups, reduce LDL-C levels in the combined training group and increase HDL-C levels in the endurance training group with no significant differences between the programmes^52^. Several factors could potentially explain the differences observed between results reported in this study and previous findings reported in our and other studies, for example, previously documented seasonal variation in cholesterol levels might affect the obtained results^24^. Dietary habits, particularly the intake of saturated fatty acids and dietary cholesterol, could also affect the lipid profile^53^. Besides, women might be more resistant to change in lipid profile when compared with men. Indeed, Ghahramanloo et al*.*^28^ found that combined training (the sum of the endurance and resistance training programme) was more effective than endurance or strength training in isolation in improving the lipid profile in young healthy men, whereas Lavie and Milani^29^ reported that 12 weeks of exercise did not significantly improve the lipid profile in elderly women. There is also some evidence that improvements in blood lipids might depend on body weight reduction^54^.

ox-LDL might play an important role in the development of atherosclerosis. It has been shown to contribute to atherosclerotic plaque formation and progression through several mechanisms, including the induction of endothelial cell activation and dysfunction, macrophage foam cell formation, and smooth muscle cell migration and proliferation^55^. Several studies also suggest that regular training may reduce ox-LDL levels. Schjerve et al.^56^ observed a decrease in ox-LDL concentrations in obese subjects after 12 weeks of strength training and moderate-intensity endurance training but not after high-intensity endurance training. Similarly, Tiainen et al*.*^57^ found that after two years of endurance-strength training, subjects with ischemic heart disease showed a decrease in ox-LDL concentrations but only in the group with a high training load. In another study conducted by the same authors, no differences in ox-LDL levels were found between the endurance training group and the control group after six months of intervention, but ox-LDL concentrations were correlated positively with body weight and negatively with VO_2_ max^58^. These results suggest that the effect of training on ox-LDL concentrations depends on body weight reduction, improvement of physical capacity and intensity of training rather than the type of exercise. In the current study, we observed no effect of endurance-strength training on ox-LDL levels, while a nonsignificant decrease of ox-LDL concentrations was detected in the endurance group (*p* = 0.0640). Nevertheless, non-differences between groups were noted. However, it should be highlighted that our study’s training programmes had similar volumes and exerted similar effects on body composition, which may partly explain the lack of differences between groups.

The effect of exercises on apolipoproteins levels remains unclear. Kokkinos et al*.*^59^ found that a 16-week moderate-intensity aerobic training programme had no effects on ApoA1 and ApoB levels in African American men with severe systemic hypertension. On the other hand, Said et al*.*^60^ observed a statistically significant increase in ApoA1 concentrations and a decrease in ApoB levels in overweight and obese women following 24 weeks of endurance and endurance-strength training. Similarly, Laaksonen et al*.*^61^ showed a statistically significant decrease in ApoB concentrations and a simultaneous increase in ApoA1 levels after 12–16 weeks of aerobic training. Park et al*.*^39^ also observed a statistically significant decrease in ApoB concentrations and an increase in ApoA1 concentrations after 24 weeks of endurance and mixed training. Our study, however, showed no effects of endurance or endurance-strength training on ApoA1 or ApoB levels. These results might be partly explained by the consistency of subjects’ lipid profiles.

PON is an HDL-associated esterase that inhibits LDL oxidative modification and suppresses the differentiation of monocytes into macrophages, which is the first stage in the development of atherosclerosis. Furthermore, PON prevents the accumulation of ox-LDL, and low PON activity increases the risk of cardiovascular disease^62^. Tas et al*.*^63^ observed a decrease in PON activity after eight weeks of continuous running, while Aicher et al*.*^64^ found that a six-month programme that included a reduced-fat and total energy diet and low-intensity exercise did not affect PON activity in obese women. On the other hand, Mahdirejei et al*.*^65^ demonstrated an increase in PON activity after four weeks of endurance training, though strength training did not affect this enzyme’s activity. It is suggested that the effect of physical activity on PON activity is associated with the *PON1-192* gene polymorphism^66^. Moreover, different age of the study population may also partly explain the differences between previous findings as this enzyme’s activity is very low at birth and increases with age^20^. The decrease in PON activity observed in our study’s endurance group may indicate increased lipid oxidation, which may be associated with a higher risk of cardiovascular disease.

Myoglobin is a marker used to monitor the effectiveness of workload on muscle tissue in exercise^67^. It has been shown that myoglobin levels may increase within 30 min of training^68^ and might remain increase even for around five days^69^. Moreover, higher levels of myoglobin after training are observed in previously untrained subjects. Besides, an increase in myoglobin serum levels correlates with exercise intensity^70^. Our study showed that not only intensity but also type of training may affect myoglobin levels. We observed that 12-week endurance training but not endurance-strength training significantly decreased myoglobin levels with significant differences noted between groups. These results might indicate a better adaptation of muscle tissue on endurance training.

Previously, it has been shown that inactivity was associated with an increased risk of developing hypertension^71^ and high BP increased the risk of stroke and ischaemic heart disease, with a reduction of BP of three mmHg associated with a 5–9% reduction in cardiac morbidity, an 8–14% reduction in stroke, and 4% reduction in all-cause mortality^72^. It seems that exercises might be effective for the prevention and treatment of hypertension^73^. Indeed, our results showed that both types of training significantly decrease BP, with endurance training being more effective than endurance-strength training in the reduction of DBP. Previously, in our pilot study, we also observed a decrease in SBP and DBP after the endurance and endurance-strength intervention but no differences between the groups^35^. Several other studies also reported that both endurance^74,75^ and endurance-strength training^76,77^ significantly decrease BP, whereas Swift et al*.*^78^ found no significant changes in BP after six months of the intervention in postmenopausal women. Similarly, Schjerve et al*.*^56^ compared the effects of strength and endurance training of moderate and high intensity in obese adults and found no changes in the SBP of all groups and a decrease in DBP in the endurance group.

The present study has several strengths and limitations. Important strengths of this study included the randomised study design and direct verification of the type, amount and intensity of training. Additionally, this study included a large number of subjects providing excellent statistical power to detect differences between training programmes. Finally, we used very strict inclusion and exclusion criteria which eliminated the impact of disrupting factors and included objective and reliable study methods (e.g., to measure body composition). The main novelty of the study is comparing the effect of endurance and endurance-strength training (both applied at the same volume, duration and intensity) in abdominally obese postmenopausal women without serious comorbidities. Moreover, this is one of the first studies, which assessed the effect of both training programmes on ox-LDL, ApoA1, ApoB and PON levels in abdominally obese postmenopausal women without severe comorbidities. Besides, the narrow age range (50–60 years) of the study participants allowed us to obtain a more homogeneous group. However, as mentioned, this study only included women with abdominal obesity, therefore, it is unknown if the training programmes would cause similar changes in men of similar age. Moreover, study participants were motivated volunteers who took part in training in a supervised setting, which limited the generalisability of the findings to the general population. Another limitation of this study is a lack of separate strength and control groups. We also did not estimate total, resting and exercise energy expenditure. Other potential confounders included differences in dietary intake and physical activity performed outside the monitoring and supervision by the researchers. Therefore, we did not know how these variables may have affected the present findings. However, all participants were instructed to maintain their normal physical activity level and eating habits. We also did not monitor the subjects after the intervention period, therefore, is unclear which type of training is more effective for the long-term reduction of the burdens of obesity.

In conclusion, both training programmes had a favourable effect on body composition in abdominally obese women but did not improve glucose and insulin homeostasis and lipid metabolism. However, we showed that only endurance training significantly decreased PON activity and reduced myoglobin levels. Besides, this type of training seems to be more effective than endurance-strength training in the reduction of DBP. Given the increasing burden of obesity, more research is needed to better understand the effect of different types of exercises on metabolic abnormalities associated with obesity.

## Methods

### Study design

The study was designed as a prospective parallel randomised trial. The study was per the standards of CONSORT^79^ and the protocol of the study was registered in the German Clinical Trials Register under the ENDOFIT acronym and with the registration number DRKS00019832, date of registration: 26/02/2020.

### Study population

Adult women, aged 50–60 years, with abdominal obesity (BMI ≥ 30 kg/m^2^, waist circumference > 80 cm, percentage of body fat ≥ 32% (the American Council on Exercise recommendation^80^) and stable body weight were recruited to the trial. The exclusion criteria included secondary obesity, previously diagnosed type 2 diabetes mellitus, coronary artery disease, stroke, congestive heart failure, arrhythmias, conduction disorders, implementation of pharmacological treatment of dyslipidaemia within the last three months, secondary hypertension or poorly controlled hypertension, liver, kidney, or thyroid diseases and cancer diagnosis. Subjects with the acute or chronic inflammatory process, connective tissue disease or arthritis, history of infection during the last month, as well as subjects with any addictions, pregnant and breastfeeding women were also excluded from the study. Study participants should not have used any dietary supplements in the three months before the study.

Volunteers were recruited to the study among patients of medical clinics and medical centres in the Greater Poland Voivodeship, in consultation with their doctors and directors of the clinics. After telephone contact, the potential subjects were screened by a physician during an inclusion visit to comply with protocol requirements.

### Ethical issues

The present study was conducted according to the guidelines in the Declaration of Helsinki. The protocol was approved by the Poznan University of Medical Sciences Bioethical Committee (refs. 219/16 and 1155/18). All study participants received information about the trial, were informed that participation was voluntary and provided written informed consent. Study participants were aware that they could withdraw at any time without providing reasons.

### Intervention

The study design and full trial protocol have been described previously^81^. Briefly, 101 women were recruited to the study and randomly divided (allocation ratio 1:1) into endurance (n = 52) and endurance-strength (n = 49) training groups. Both groups performed 36 supervised endurance or endurance-strength training, three times per week during the three-month intervention. Subjects who completed less than 29 training were excluded from the analysis. The training programmes consisted of five minutes of warm-up at low intensity, 45 min of endurance exercises in the endurance group or 20 min of strength exercises and 25 min of endurance exercises in the endurance-strength group, five minutes of cycling without load and five minutes of closing stretching. The endurance exercises were performed on cycle ergometers (Schwinn Evolution, Schwinn Bicycle Company, Boulder, Colorado, USA) at an intensity between 50–70% of maximum heart rate (HR max). The strength component involved exercises with a barbell and a gymnastic ball at 50–60% of one-repetition maximum (the maximum load that subject can lift). The intensity of both types of training was individually selected for each subject and did not change during the intervention. The strength training was repeated in a series, with the number of repetitions dependent on the subjects’ muscle strength and systematically increased with the increase in the subjects’ muscle strength. The goal number of repetitions per set was 16 in barbell curls and 30 in barbell squats. Between the series, short pauses were taken (10–15 s), during which subjects conducted isometric exercises. Aside from the training, all subjects were instructed to maintain their usual physical activity level and eating habits. No deviation from the study protocol was observed.

Our previous pilot study also assessed the effect of 12-week endurance and endurance-strength training programmes on body composition, BP and selected biochemical parameters. However, the pilot study included a small number of subjects of heterogeneous age (28–62 years)^35,52^. Due to the negative effect of training on bone health (data not published) observed in our pilot trial, here we slightly modified endurance training including cycling with a load.

### Outcomes

The primary outcomes of the study were the effect of endurance and endurance-strength training on endothelial parameters^81^. Here, we reported the effect on secondary outcomes, including body composition (FM, VAT, ALMI and LMI), biochemical markers (glucose and insulin homeostasis and lipid metabolism), BP (SBP and DBP). All outcomes were measured and collected at the Poznan University of Medical Sciences before and after the intervention period. Methods used to measure the outcomes were identical in both groups.

### Anthropometric parameters and body composition

After at least eight hours of overnight fasting, the following anthropometric parameters were measured body height, body weight, waist and hip circumferences. BMI was calculated and body composition was assessed using a dual-energy X-ray absorptiometry (DEXA) method with the application of the Hologic Discovery DEXA system (Bedford, MA, USA). Based on the examination, FM and FFM for total body and individual parts of the body (arms, trunk, legs, head), male (android) and female (gynoid) areas were measured. VAT, ALMI and LMI were also assessed. During all measurements, participants were dressed in light clothing and were barefoot.

### Blood pressure

BP was measured during the recruitment visit and on the last visit according to guidelines of the European Society of Hypertension^82^. The average of three measurements was used for statistical analysis.

### Biochemical measurements

Pre- and seven days post-intervention period fasting blood samples were collected for routine analysis of glucose and insulin homeostasis (glucose, insulin, HbA1c and IGF-1 levels) and lipid metabolism (TC, LDL-C, HDL-C, TG), ox-LDL, apolipoproteins (ApoA1, ApoB), and PON levels. Besides, myoglobin levels were assessed. HOMA-IR, QUICKI and ApoB/ApoA1 ratio were also calculated. All parameters were measured by standard methods as described previously^81^. Glucose levels were assessed by the enzymatic method with hexokinase, insulin levels were analysed using the electrochemiluminescence method and HbA1c levels were measured by the turbidimetric immunoinhibitory method in hemolysate prepared from the blood. TC, HDL-C and TG concentrations were assessed using the enzymatic colorimetric method, while LDL-C levels were calculated from the Friedewald formula. The following parameters were measured using the immunoenzymatic method: IGF-1 (IGF-1 600 ELISA kit, DRG Instruments GmbH, Germany), ox-LDL (Human ox-LDL ELISA kit, SunRed, China) and myoglobin (Myoglobin ELISA kit, DRG Instruments GmbH, Germany). Finally, the nephelometric method was used to analysed ApoA1 and ApoB levels.

### Randomisation and blinding

Randomisation was performed via computer software (Random Allocation Software, Isfahan, Iran) by an independent researcher. Stratified randomisation was used and a computer-generated randomisation list was generated. The subjects were stratified according to age, body weight, BMI and waist circumference. The allocation sequence was concealed until subjects were enrolled to interventions. After randomisation, study participants, health professionals and other research staff involved in the trial were not blinded. However, study team members who assessed the outcomes, prepared the database and performed the statistical analysis were not aware of allocation.

### Minimum sample size

The minimum sample size was calculated based on the changes in eNOS levels (endothelial function marker which was the primary outcome of the study) reported previously in our pilot study^47^. The G*Power 3.1.9.2 software (University of Kiel, Kiel, Germany) was used. To obtain a power of 80% (α = 0.05, β = 0.2) at least 40 subjects per group should be recruited. Assuming that 20% of subjects may withdraw from the study, a minimum of 48 women per group were needed. Moreover, we also performed the calculations based on changes in LDL-C levels (secondary outcome) reported previously by Rossi et al.^25^. According to the calculations, at least 41 subjects should be included in each group.

### Statistical analysis

Statistical analysis was performed using the STATISTICA 13.0 software (TIBCO Software Inc., Palo Alto, USA). A two-sided *p*-value ≤ 0.05 was regarded as significant. We used the Shapiro–Wilk test to assess the normal distribution of data. Data are presented as medians and interquartile range (IQR; Q1–Q3) as well as means and standard deviations (SD) with 95% CI. Results were also expressed as changes between pre- and post-intervention values (Δ value at third month). Comparisons between groups were conducted using the Mann–Whitney test and the Wilcoxon test was used to analyse the differences between pre- and post-intervention values. The effectiveness of exercise programmes was examined by comparing the mean difference of changes in each variable using the ANCOVA test, adjusted for the baseline measures as a covariate. Data with non-normal distribution was normalised before the analysis. For ease of interpretation, data was back-transformed.

## Data Availability

The datasets generated during and/or analysed during the current study are not publicly available due to the disagreement of the study participants but are available from the corresponding author on reasonable request.

## References

[1] World Health Organization. *Waist circumference and waist-hip ratio: report of a WHO expert consultation*. *World Health Organization* (2008).

[2] Zhang C, Rexrode KM, van Dam RM, Li TY, Hu FB (2008). Abdominal obesity and the risk of all-cause, cardiovascular, and cancer mortality: sixteen years of follow-up in US women. Circulation.

[3] Cameron AJ, Zimmet PZ (2008). Expanding evidence for the multiple dangers of epidemic abdominal obesity. Circulation.

[4] Carmienke S (2013). General and abdominal obesity parameters and their combination in relation to mortality: a systematic review and meta-regression analysis. Eur. J. Clin. Nutr..

[5] Peeters A (2003). Obesity in adulthood and its consequences for life expectancy: a life-table analysis. Ann. Intern. Med..

[6] Yumuk V (2015). European guidelines for obesity management in adults. Obes. Facts.

[7] Garvey WT (2016). American Association of Clinical Endocrinologists and American College of Endocrinology comprehensive clinical practice guidelines for medical care of patients with obesity. Endocr. Pract..

[8] Jensen MD (2014). 2013 AHA/ACC/TOS guideline for the management of overweight and obesity in adults: a report of the American College of Cardiology/American Heart Association Task Force on Practice Guidelines and The Obesity Society. Circulation.

[9] Ryan DH, Kahan S (2018). Guideline recommendations for obesity management. Med. Clin. N. Am..

[10] Dipietro L (2020). Physical activity and cardiometabolic risk factor clustering in young adults with obesity. Med. Sci. Sports Exerc..

[11] Amaro-Gahete FJ (2019). Exercise training as a treatment for cardiometabolic risk in sedentary adults: are physical activity guidelines the best way to improve cardiometabolic health? The FIT-AGEING randomized controlled trial. J. Clin. Med..

[12] Ritti-Dias RM (2017). Self-initiated changes in physical activity levels improve cardiometabolic profiles: a longitudinal follow-up study. Nutr. Metab. Cardiovasc. Dis..

[13] Donnelly JE (2009). American College of Sports Medicine Position Stand. Appropriate physical activity intervention strategies for weight loss and prevention of weight regain for adults. Med. Sci. Sports Exerc..

[14] Jakicic JM (2001). American College of Sports Medicine position stand. Appropriate intervention strategies for weight loss and prevention of weight regain for adults. Med. Sci. Sports Exerc..

[15] Fogelholm M, Stallknecht B, van Baak M (2006). ECSS position statement: exercise and obesity. Eur. J. Sport Sci..

[16] Lemes ÍR, Turi-Lynch BC, Cavero-Redondo I, Linares SN, Monteiro HL (2018). Aerobic training reduces blood pressure and waist circumference and increases HDL-c in metabolic syndrome: a systematic review and meta-analysis of randomized controlled trials. J. Am. Soc. Hypertens.

[17] Oliver-Martínez PA, Ramos-Campo DJ, Martínez-Aranda LM, Martínez-Rodríguez A, Rubio-Arias JÁ (2020). Chronic effects and optimal dosage of strength training on SBP and DBP: a systematic review with meta-analysis. J. Hypertens.

[18] Wewege MA, Thom JM, Rye KA, Parmenter BJ (2018). Aerobic, resistance or combined training: a systematic review and meta-analysis of exercise to reduce cardiovascular risk in adults with metabolic syndrome. Atherosclerosis.

[19] Ashton RE (2020). Effects of short-term, medium-term and long-term resistance exercise training on cardiometabolic health outcomes in adults: systematic review with meta-analysis. Br. J. Sports Med..

[20] Costa RR (2019). Effect of strength training on lipid and inflammatory outcomes: systematic review with meta-analysis and meta-regression. J. Phys. Act Health.

[21] Ho SS, Dhaliwal SS, Hills AP, Pal S (2012). The effect of 12 weeks of aerobic, resistance or combination exercise training on cardiovascular risk factors in the overweight and obese in a randomized trial. BMC Public Health.

[22] Sanal E, Ardic F, Kirac S (2013). Effects of aerobic or combined aerobic resistance exercise on body composition in overweight and obese adults: gender differences. A randomized intervention study. Eur. J. Phys. Rehabil. Med..

[23] AbouAssi H (2015). The effects of aerobic, resistance, and combination training on insulin sensitivity and secretion in overweight adults from STRRIDE AT/RT: a randomized trial. J. Appl. Physiol..

[24] Boardley D, Fahlman M, Topp R, Morgan AL, McNevin N (2007). The impact of exercise training on blood lipids in older adults. Am. J. Geriatr. Cardiol..

[25] Rossi FE (2016). Combined training (aerobic plus strength) potentiates a reduction in body fat but demonstrates no difference on the lipid profile in postmenopausal women when compared with aerobic training with a similar training load. J. Strength Cond. Res..

[26] Schwingshackl L, Dias S, Strasser B, Hoffmann G (2013). Impact of different training modalities on anthropometric and metabolic characteristics in overweight/obese subjects: a systematic review and network meta-analysis. PLoS ONE.

[27] Donnelly JE (2003). Effects of a 16-month randomized controlled exercise trial on body weight and composition in young, overweight men and women: the midwest exercise trial. Arch. Intern. Med..

[28] Ghahramanloo E, Midgley AW, Bentley DJ (2009). The effect of concurrent training on blood lipid profile and anthropometrical characteristics of previously untrained men. J. Phys. Act Health.

[29] Lavie CJ, Milani RV (1997). Benefits of cardiac rehabilitation and exercise training in elderly women. Am. J. Cardiol..

[30] Dupuit M (2020). Effect of high intensity interval training on body composition in women before and after menopause: a meta-analysis. Exp. Physiol..

[31] el Khoudary SR (2020). Menopause transition and cardiovascular disease risk: implications for timing of early prevention: a scientific statement from the American Heart Association. Circulation.

[32] Zhu D (2018). Relationships between intensity, duration, cumulative dose, and timing of smoking with age at menopause: a pooled analysis of individual data from 17 observational studies. PLoS Med..

[33] Lloyd-Jones DM (2006). Prediction of lifetime risk for cardiovascular disease by risk factor burden at 50 years of age. Circulation.

[34] Rodgers JL (2019). Cardiovascular risks associated with gender and aging. J. Cardiovasc. Dev. Dis..

[35] Skrypnik D (2015). Effects of endurance and endurance strength training on body composition and physical capacity in women with abdominal obesity. Obes. Facts.

[36] Sillanpää E (2009). Body composition, fitness, and metabolic health during strength and endurance training and their combination in middle-aged and older women. Eur. J. Appl. Physiol..

[37] Church TS (2010). Effects of aerobic and resistance training on hemoglobin A1c levels in patients with type 2 diabetes. JAMA.

[38] Marzolini S, Oh PI, Brooks D (2012). Effect of combined aerobic and resistance training versus aerobic training alone in individuals with coronary artery disease: a meta-analysis. Eur. J. Prev. Cardiol..

[39] Park S-K (2003). The effect of combined aerobic and resistance exercise training on abdominal fat in obese middle-aged women. J. Physiol. Anthropol. Appl. Human.

[40] Geer EB, Shen W (2009). Gender differences in insulin resistance, body composition, and energy balance. Gend. Med..

[41] Henriksen EJ (2002). Invited review: effects of acute exercise and exercise training on insulin resistance. J. Appl. Physiol..

[42] Holten MK (2004). Strength training increases insulin-mediated glucose uptake, GLUT4 content, and insulin signaling in skeletal muscle in patients with type 2 diabetes. Diabetes.

[43] Azarbayjani M, Abedi B, Peeri M, Stannard SR (2014). Effects of combined aerobic and resistant training on lipid profile and glycemic control in sedentary men. Int. Med. J..

[44] Marson EC, Delevatti RS, Prado AKG, Netto N, Kruel LFM (2016). Effects of aerobic, resistance, and combined exercise training on insulin resistance markers in overweight or obese children and adolescents: a systematic review and meta-analysis. Prev. Med..

[45] Sigal RJ (2007). Effects of aerobic training, resistance training, or both on glycemic control in type 2 diabetes: a randomized trial. Ann. Intern. Med..

[46] Davidson LE (2009). Effects of exercise modality on insulin resistance and functional limitation in older adults. Arch. Intern. Med..

[47] Mann S, Beedie C, Jimenez A (2014). Differential effects of aerobic exercise, resistance training and combined exercise modalities on cholesterol and the lipid profile: review, synthesis and recommendations. Sports Med..

[48] Baigent C (2010). Efficacy and safety of more intensive lowering of LDL cholesterol: a meta-analysis of data from 170,000 participants in 26 randomised trials. Lancet.

[49] Roger VL (2012). Heart disease and stroke statistics-2012 update: a report from the American Heart Association. Circulation.

[50] World Health Organization. Chapter 1: Burden : mortality, morbidity and risk factors. In: *Global status report on non-communicable diseases 2010* 9–31 (2011). doi: ISBN 978 92 4 068645 8.

[51] Schwingshackl L, Missbach B, Dias S, König J, Hoffmann G (2014). Impact of different training modalities on glycaemic control and blood lipids in patients with type 2 diabetes: a systematic review and network meta-analysis. Diabetologia.

[52] Ratajczak M (2019). Effects of endurance and endurance-strength training on endothelial function in women with obesity: a randomized trial. Int. J. Environ. Res. Public Health.

[53] Siri-Tarino PW, Krauss RM (2016). Diet, lipids, and cardiovascular disease. Curr. Opin. Lipidol..

[54] Leon AS, Sanchez OA (2001). Response of blood lipids to exercise training alone or combined with dietary intervention. Med. Sci. Sports Exerc..

[55] Pirillo A, Norata G, Catapano A (2013). LOX-1, OxLDL, and atherosclerosis. Mediators Inflamm.

[56] Schjerve IE (2008). Both aerobic endurance and strength training programmes improve cardiovascular health in obese adults. Clin. Sci. (Lond).

[57] Tiainen S (2018). Effects of a two-year home-based exercise training program on oxidized LDL and HDL lipids in coronary artery disease patients with and without type-2 diabetes. Antioxidants (Basel).

[58] Tiainen S, Luoto R, Ahotupa M, Raitanen J, Vasankari T (2016). 6-mo aerobic exercise intervention enhances the lipid peroxide transport function of HDL. Free Radic. Res..

[59] Kokkinos PF (1998). Effects of moderate intensity exercise on serum lipids in African-American men with severe systemic hypertension. Am. J. Cardiol..

[60] Said M, Lamya N, Olfa N, Hamda M (2017). Effects of high-impact aerobics vs. low-impact aerobics and strength training in overweight and obese women. J. Sports Med. Phys. Fitness.

[61] Laaksonen DE (2000). Aerobic exercise and the lipid profile in type 1 diabetic men: a randomized controlled trial. Med. Sci. Sports Exerc..

[62] Lioudaki S (2017). Paraoxonase-1: characteristics and role in atherosclerosis and carotid artery disease. Curr. Vasc. Pharmacol..

[63] Tas M, Zorga E, Yaman M (2012). Comparison of the effects of different training methods on arylesterase activity and paraoxonase activity levels in hot environment. Online J. Recreation Sport.

[64] Aicher BO (2012). Diet-induced weight loss in overweight or obese women and changes in high-density lipoprotein levels and function. Obesity.

[65] Mahdirejei TA (2015). A comparative study of the effects of endurance and resistance exercise training on PON1 and lipid profile levels in obese men. Sport Sci. Health.

[66] Nalcakan GR (2016). Effects of aerobic training on serum paraoxonase activity and its relationship with PON1-192 phenotypes in women. J. Sport Health Sci..

[67] Speranza L (2007). Plasmatic markers of muscular stress in isokinetic exercise. J. Biol. Regul. Homeost Agents.

[68] Ascensão A (2008). Biochemical impact of a soccer match - analysis of oxidative stress and muscle damage markers throughout recovery. Clin. Biochem..

[69] Neubauer O, König D, Wagner KH (2008). Recovery after an Ironman triathlon: sustained inflammatory responses and muscular stress. Eur. J. Appl. Physiol..

[70] Sabriá M, Ruibal A, Rey C, Foz M, Domenech FM (1983). Influence of exercise on serum levels of myoglobin measured by radioimmunoassay. Eur. J. Nucl. Med..

[71] Bouaziz W, Schmitt E, Kaltenbach G, Geny B, Vogel T (2015). Health benefits of endurance training alone or combined with diet for obese patients over 60: a review. Int. J. Clin. Pract..

[72] Cook NR, Cohen J, Hebert PR, Taylor JO, Hennekens CH (1995). Implications of small reductions in diastolic blood pressure for primary prevention. Arch. Intern. Med..

[73] Smart NA (2020). Physical activity to prevent and treat hypertension: a systematic review. Med. Sci. Sports Exerc..

[74] Monteiro, L. Z., Vaz Fiani, C. R., de Freitas, M. C. F., Zanetti, M. L. & Foss, M. C. Redução da pressão arterial, do imc e da glicose após treinamento aeróbico em idosas com diabete tipo 2. *Arq. Bras. Cardiol.* **95**, 563–570 (2010). 10.1590/s0066-782x2010005000135 20922265

[75] Braz NFT (2012). Influence of aerobic training on cardiovascular and metabolic parameters in ederly hypertensive women. Int. J. Prev. Med..

[76] Berent R (2011). Resistance training dose response in combined endurance-resistance training in patients with cardiovascular disease: a randomized trial. Arch. Phys. Med. Rehabil..

[77] de Moraes WM (2012). Programa de exercícios físicos baseado em frequência semanal mínima: efeitos na pressão arterial e aptidão física em idosos hipertensos. Rev. Bras. Fisioter.

[78] Swift DL (2012). Exercise training and habitual physical activity: a randomized controlled trial. Am. J. Prev. Med..

[79] Moher D (2010). CONSORT 2010 explanation and elaboration: updated guidelines for reporting parallel group randomised trials. BMJ.

[80] American Council on Exercise. Percent Body Fat Calculator. https://www.acefitness.org/education-and-resources/lifestyle/tools-calculators/percent-body-fat-calculator/ (2020).

[81] Jamka M (2020). Comparison of the effects of endurance and endurance-strength training programmes on the level of endothelial dysfunction in women with abdominal obesity: study protocol for a randomised controlled trial. J. Med. Sci..

[82] Williams B (2018). 2018 ESC/ESH guidelines for the management of arterial hypertension. Eur. Heart J..

[83] Jamka M (2021). Endurance training depletes antioxidant system but does not affect endothelial functions in women with abdominal obesity: a randomized trial with a comparison to endurance-strength training. J. Clin. Med..

